# Sodium intake in Germany

**DOI:** 10.17886/RKI-GBE-2016-041

**Published:** 2016-12-14

**Authors:** Stefanie Klenow, Gert B.M. Mensink

**Affiliations:** Robert Koch Institute, Department for Epidemiology and Health Monitoring, Berlin, Germany

**Keywords:** NUTRITION, SODIUM, HEALTH SURVEY, DEGS1, GERMANY

## Abstract

For many years, a high sodium intake has been discussed as a potential risk factor in the development of hypertension and, consequently, cardiovascular diseases. As part of the German Health Interview and Examination Survey for Adults (DEGS1), which was conducted between 2008 and 2011, sodium excretion in casual urine samples was used as a biomarker to measure sodium intake. DEGS1 observed that the median daily sodium intake of women (3.4 g) as well as men (4.0 g) exceeds the levels recommended by German and international organisations. Among other factors, men’s higher sodium intake could be explained by their higher energy intake. In addition, DEGS1 demonstrates an association between women’s sodium intake and age; however, no equivalent correlation was identified for men. Furthermore, although high socio-economic status is associated with lower sodium intake in men, no comparable correlation was observed among women.

## Introduction

Sodium is an essential nutrient with important functions in the body [1]. It is a component of table salt, which is added to foods during processing, preparation, and immediately before eating. As such, processed foods, and especially bread, prepared meat products, and dairy products such as cheese, are the primary sources of sodium [2, 3].

A high sodium intake is associated with a risk of high blood pressure (hypertension) and is thereby indirectly related to the development of cardiovascular disease [4–10]. However, not all people respond with elevated blood pressure to high salt intake (salt sensitivity) [11, 12]. Further negative effects of a high sodium intake have also been discussed: these include a possible higher risk of stomach cancer and osteoporosis [13].

In order to compensate for daily losses of sodium, a minimum intake of 0.55 g per day for adults and young people is recommended [1]. The German Nutrition Society (DGE) has defined a guidance level of up to 6 g per day for table salt [14]. This is comparable to a teaspoon of salt and corresponds to a daily sodium intake of about 2.4 g.

A representative and regular assessment of the population’s sodium intake in Germany is an important means of assessing and developing practical approaches for the future.

## Indicator

The amount of sodium that is excreted throughout the day is assumed to roughly correspond to a person’s daily sodium intake; as such, the amount of sodium measured in the urine constitutes a suitable biomarker to measure sodium intake. Casual urine samples were collected as part of the German Health Interview and Examination Survey for Adults (DEGS1), which was conducted by the Robert Koch Institute between 2008 and 2011 [15]. The measured sodium concentrations were converted to estimates of daily sodium excretion based on creatinine concentrations [15]. The results on sodium intake are analysed according to gender, age, and socio-economic status.

## Reflection of the results

The German adult population has a median sodium intake of 3.7 g per day. The median intake of women (3.4 g) is lower than men (4.0 g) (Table 1). This means that 50% of the adult population in Germany has a daily sodium intake that is higher than this level. Sodium intake of 25% of women is 5.0 g per day or more; 25% of men have a sodium intake of 5.7 g per day or more (Table 1, 75^th^ percentile); 5% of women consume more than 8.1 g of sodium per day; and 5% of men consume more than 8.8 g of sodium per day (Table 1, 95^th^ percentile). The mean estimated daily sodium intake (3.8 g for women and 4.5 g for men) is higher than the corresponding median values. Men’s higher sodium intake could be explained by their higher energy intake. In terms of units of energy, men and women in Germany have a similar sodium intake [16].

Women’s sodium intake does increase until the 40-to-59 age group and decreases slightly in the older age groups. No such age-related trends have been observed among men.

There is no association between women’s socio-economic status and sodium intake (Table 2). In contrast, men with a high socio-economic status consume slightly less sodium than men with a mid to low socio-economic status (Table 2).

Estimates of sodium intake in Germany based on sodium excretions in urine are consistently higher than those gained from dietary surveys [2, 3, 11, 17, 18]. Differences in methodology probably account for the discrepancies between the results. The German National Nutrition Survey II determined sodium intake using dietary history interviews (software: DISHES) and two 24-hour recalls [3, 17]. The study found women’s median daily sodium intake to be 2.4 g and 1.9 g depending on the specific method and men’s median sodium intake at 3.2 g and 2.8 g. The German National Health Interview and Examination Survey 1998 (GNHIES98), which also used DISHES to estimate sodium intake, estimated the daily median sodium intake to be 2.2 g for women and 3.0 g for men [2, 11].

The estimated international average daily sodium intake varies between 2.6 g and 4.8 g and the mean value (calculated using all of the included studies) is 3.7 g [19]. Compared with international levels, the mean daily sodium intake in Germany – 4.1 g – (estimated using sodium excretion) is in the mid to high range.

Compared with recommendations made by national and international organisations, large parts of the German population consume too much sodium: 73% of women and 80% of men in Germany exceed the guidance level defined by the German Nutrition Society [14] of up to 6 g of table salt per day (equivalent to 2.4 g of sodium). Furthermore, 80% of women and 86% of men in Germany exceed the World Health Organization’s (WHO) [20] recommendation of a sodium intake of less than 2 g per day.

Since the daily sodium intake in many EU member states is higher than recommended levels, the WHO’s Action plan for implementation of the European Strategy for the Prevention and Control of Noncommunicable Diseases 2012–2016 declared the reduction of sodium intake to be one of its five priority areas for intervention [21].

## Key statements

- Based on German and international recommendations, sodium intake of large parts of the German population is too high.
- Men tend to have a higher sodium intake than women; this is linked to men’s higher dietary energy intake.
- Women’s sodium intake is lower among younger and among the highest age groups.
- Men with a high socio-economic status tend to have lower sodium intakes.

## Figures and Tables

**Table 1:** Estimated sodium intake (percentile) for 18- to 79-year-olds according to gender and age (n=3,626 women, n=3,333 men) Source: DEGS1 (2008–2011)

	P5 g/day	P10 g/day	P25 g/day	Median g/day	P75 g/day	P90 g/day	P95 g/day
**Women (all)**	**1.1**	**1.5**	**2.3**	**3.4**	**5.0**	**6.7**	**8.1**
**Age**							
18 – 29 years	1.0	1.3	2.0	2.9	4.2	5.9	7.0
30 – 39 years	1.3	1.5	2.3	3.3	4.7	6.4	7.8
40 – 49 years	1.3	1.6	2.6	3.8	5.2	7.1	8.5
50 – 59 years	1.1	1.5	2.4	3.7	5.4	7.0	8.0
60 – 69 years	1.1	1.5	2.1	3.4	4.8	6.7	7.9
70 – 79 years	0.9	1.3	2.1	3.1	5.0	6.6	8.6
**Men (all)**	**1.2**	**1.7**	**2.7**	**4.0**	**5.7**	**7.6**	**8.8**
**Age**							
18 – 29 years	1.5	1.8	2.6	3.9	6.0	7.9	8.7
30 – 39 years	1.1	1.8	2.9	4.2	6.1	8.2	9.2
40 – 49 years	1.0	1.5	2.6	3.8	5.3	6.9	8.6
50 – 59 years	1.3	1.9	2.7	4.1	5.9	7.8	9.0
60 – 69 years	1.3	1.7	2.7	4.1	5.7	7.6	9.6
70 – 79 years	1.2	1.7	2.6	3.9	5.5	7.6	8.6
**Total**	**1.2**	**1.6**	**2.4**	**3.7**	**5.3**	**7.2**	**8.6**

P = percentile

**Table 2:** Estimated sodium intake (median) among 18- to 79-year-olds according to gender and socio-economic status (n=3,602 women, n=3,309 men) Source: DEGS1 (2008–2011)

	Women g/day	Men g/day
**Socio-economic status**		
Low	3.4	4.0
Medium	3.5	4.1
High	3.4	3.7

## References

[1] Deutsche Gesellschaft für Ernährung, Österreichische Gesellschaft für Ernährung, Schweizerische Gesellschaft für Ernährungsforschung, Schweizerische Vereinigung für Ernährung (Hrsg) (2015) D-A-CH Referenzwerte für die Nährstoffzufuhr. DGE, ÖGE, SGE, Bonn

[2] Mensink GB Burger M Beitz R (2002) Was essen wir heute? Ernährungsverhalten in Deutschland. RKI, Berlin

[3] Max Rubner-Institut, Bundesforschungsinstitut für Ernährung und Lebensmittel (2008) Nationale Verzehrsstudie II – Die bundesweite Befragung zur Ernährung von Jugendlichen und Erwachsenen: Ergebnisbericht Teil 2. MRI, Karlsruhe

[4] He FJ MacGregor GA (2004) Effect of longer-term modest salt reduction on blood pressure. Cochrane Database Syst Rev (3):CD004937 15266549 10.1002/14651858.CD004937

[5] Hooper L Bartlett C Davey SG (2004) Advice to reduce dietary salt for prevention of cardiovascular disease. Cochrane Database Syst Rev (1):CD003656 14974027 10.1002/14651858.CD003656.pub2 PMC12160996

[6] He FJ MacGregor GA (2011) Salt reduction lowers cardiovascular risk: meta-analysis of outcome trials. The Lancet 378(9789):380-382 10.1016/S0140-6736(11)61174-4 21803192

[7] Mente A O’Donnell MJ Yusuf S (2014) The population risks of dietary salt excess are exaggerated. Can J Cardiol 30(5):507-512 24786440 10.1016/j.cjca.2014.02.003

[8] O’Donnell M Mente A Rangarajan S (2014) Urinary sodium and potassium excretion, mortality, and cardiovascular events. N Engl J Med 371(7):612-623 25119607 10.1056/NEJMoa1311889

[9] Strazzullo P D’Elia L Kandala NB (2009) Salt intake, stroke, and cardiovascular disease: meta-analysis of prospective studies. BMJ 339:b4567 19934192 10.1136/bmj.b4567 PMC2782060

[10] Stamler J (1997) The INTERSALT Study: background, methods, findings, and implications. Am J Clin Nutr 65(2 Suppl):626S-642S 9022559 10.1093/ajcn/65.2.626S

[11] Bundesinstitut für Risikobewertung (2011) Blutdrucksenkung durch weniger Salz in Lebensmitteln. Stellungnahme Nr. 007/2012 des BfR, MRI und RKI vom 19. Oktober 2011. BfR, Berlin, P. 21

[12] Pilic L Pedlar CR Mavrommatis Y (2016) Salt-sensitive hypertension: mechanisms and effects of dietary and other lifestyle factors. Nutr Rev 74(10):645-658 27566757 10.1093/nutrit/nuw028

[13] Cappuccio FP (2013) Cardiovascular and other effects of salt consumption. Kidney Int Suppl (2011) 3(4):312-315 25019010 10.1038/kisup.2013.65 PMC4089690

[14] Strohm D Boeing H Leschik-Bonnet E (2016) Speisesalzzufuhr in Deutschland, gesundheitliche Folgen und resultierende Handlungsempfehlungen. Ernährungs Umschau 63(3):62-70

[15] Klenow S Thamm M Mensink GBM (2016) Sodium intake in Germany estimated from sodium excretion measured in spot urine samples. BMC Nutrition 2(1)

[16] Simmet A Mensink GBM Stroebele N (2012) Association of dietary sodium intake and blood pressure in the German population. J Public Health 20(6):621-630

[17] Deutsche Gesellschaft für Ernährung (2012) 12. Ernährungsbericht 2012. Deutsche Gesellschaft für Ernährung e.V. (DGE), Bonn

[18] Hartmann BM Grotz A Stang K (2011) Die aktuelle Version 3.01 des Bundeslebensmittelschlüssels (BLS): Neuerungen und Auswirkungen. Proceedings of the German Nutrition Society, Vol 15

[19] McCarron DA Kazaks AG Geerling JC (2013) Normal range of human dietary sodium intake: a perspective based on 24-hour urinary sodium excretion worldwide. Am J Hypertens 26(10):1218-1223 23978452 10.1093/ajh/hpt139

[20] World Health Organization (2012) Guideline: Sodium Intake for Adults and Children. WHO, Geneva 23658998

[21] World Health Organization (2012) Aktionsplan zur Umsetzung der Europäischen Strategie zur Prävention und Bekämpfung nichtübertragbarer Krankheiten (2012–2016). www.euro.who.int/__data/assets/pdf_file/0011/174629/e96638-Ger.pdf?ua=1 (As at 27.9.2016)

[22] Klenow S Mensink GBM (2016) Natrium. The German Nutrition Society (DGE), 13^th^ Nutrition Report. Bonn, P. 52-57

